# No mass image formation of metastatic neuroendocrine tumors to the liver on ultrasound: A case report

**DOI:** 10.1016/j.radcr.2025.04.121

**Published:** 2025-05-22

**Authors:** Hiroki Yamamoto, Shoji Oura, Hiroshi Shintani

**Affiliations:** Department of Surgery, Kishiwada Tokushukai Hospital, Kishiwada-city, Japan

**Keywords:** Liver metastasis, Neuroendocrine tumor, No tumor depiction, Ultrasound

## Abstract

A 65-year-old woman showed abnormal positron emission tomography findings in the liver after breast cancer surgery. Computed tomography showed a hepatic mass with faint ring enhancement. Magnetic resonance imaging (MRI) of the liver mass showed low signals on T1-weighted images, faint high signals on fat-suppressed T2-weighted images, nominal enhancement both on early and late phase images, and distinct low signals on hepatobiliary images. Ultrasound, however, could not depict any mass images in the liver. The patient underwent diagnostic endoscopic laparotomy, revealing a visible liver tumor through the liver surface in the segment 5. Laparoscopic ultrasound was also unable to depict any tumor images even by directly placing the ultrasound probe just on the exposed tumor. Pathological study of the resected mass showed that a well-circumscribed mass was composed of atypical cells growing in solid and trabecular fashions with little nuclear atypia, focal shelf-like arrangement of nuclei, a fine vascular network, small interstitial components between tumor cell clusters, and no peri-tumoral inflammatory cells/fibrous components, and had 3 daughter nodules. In addition, CD56, synaptophysin, and chromogranin positivities of the tumors led to the diagnosis of metastatic neuroendocrine tumors (NET). Diagnostic physicians should note that ultrasound may fail to show mass images even of well-circumscribed metastatic NETs to the liver.

## Introduction

Neuroendocrine tumors (NETs) are rare solid malignancies [1]. Functional NETs are often detected in their early phases due to their characteristic symptoms, whereas nonfunctional NETs are generally not detected until the tumors grow large enough to invade or compress surrounding organs or tissues and develop some symptoms [2].

Endocrine cells are present throughout the body, and therefore give rise to NETs in any organs and tissues. Approximately 70% of NET cases, however, are observed in the gastrointestinal tract, especially most often in the pancreas [3]. Due to these characteristic predilection sites, NETs most frequently metastasize to the liver. However, even when liver metastasis is detected, the primary NETs are sometimes not detected on any images including positron emission tomography/computed tomography (PET/CT) scans due to their indolent biology.

We herein report a metastatic NET to the liver which could not de depicted even by directly placing the ultrasound probe just on the exposed liver tumor.

## Case presentation

**Fig. 1:**
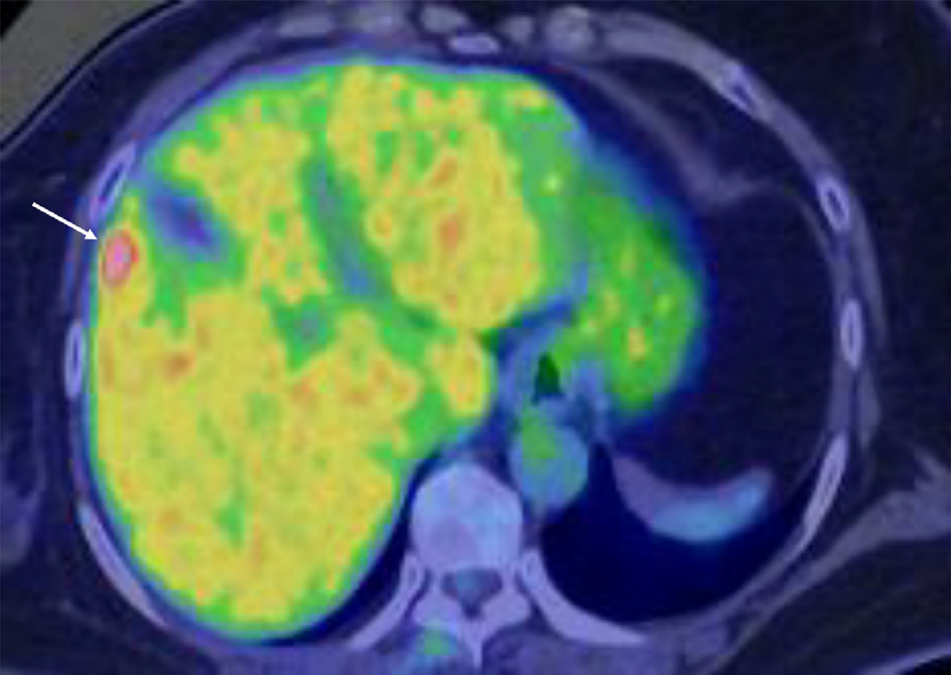
Positron emission tomography / computed tomography (PET/CT) findings. PET/CT showed an avid accumulation (arrow) of fluorodeoxyglucose in the liver segment 5.

**Fig. 2:**
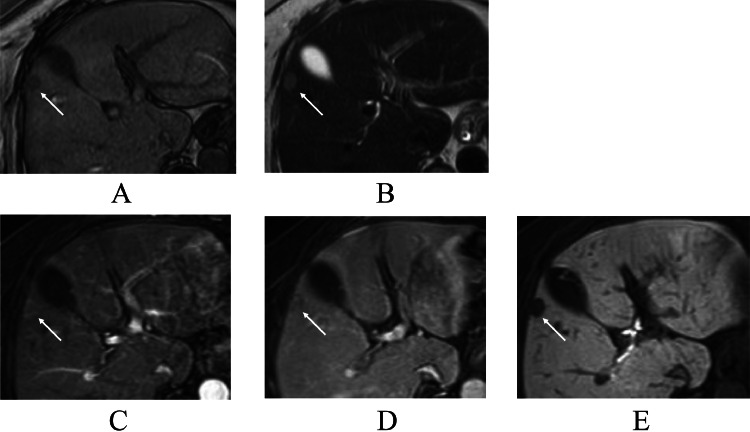
Magnetic resonance imaging (MRI) findings. MRI of the tumor (arrows) showed low signals on T1-weighted images (A), faint high signals on T2-weighted images (B), nominal enhancement both on early and late phase images (C and D), and very low signals on hepatobiliary phase images (E).

**Fig. 3:**
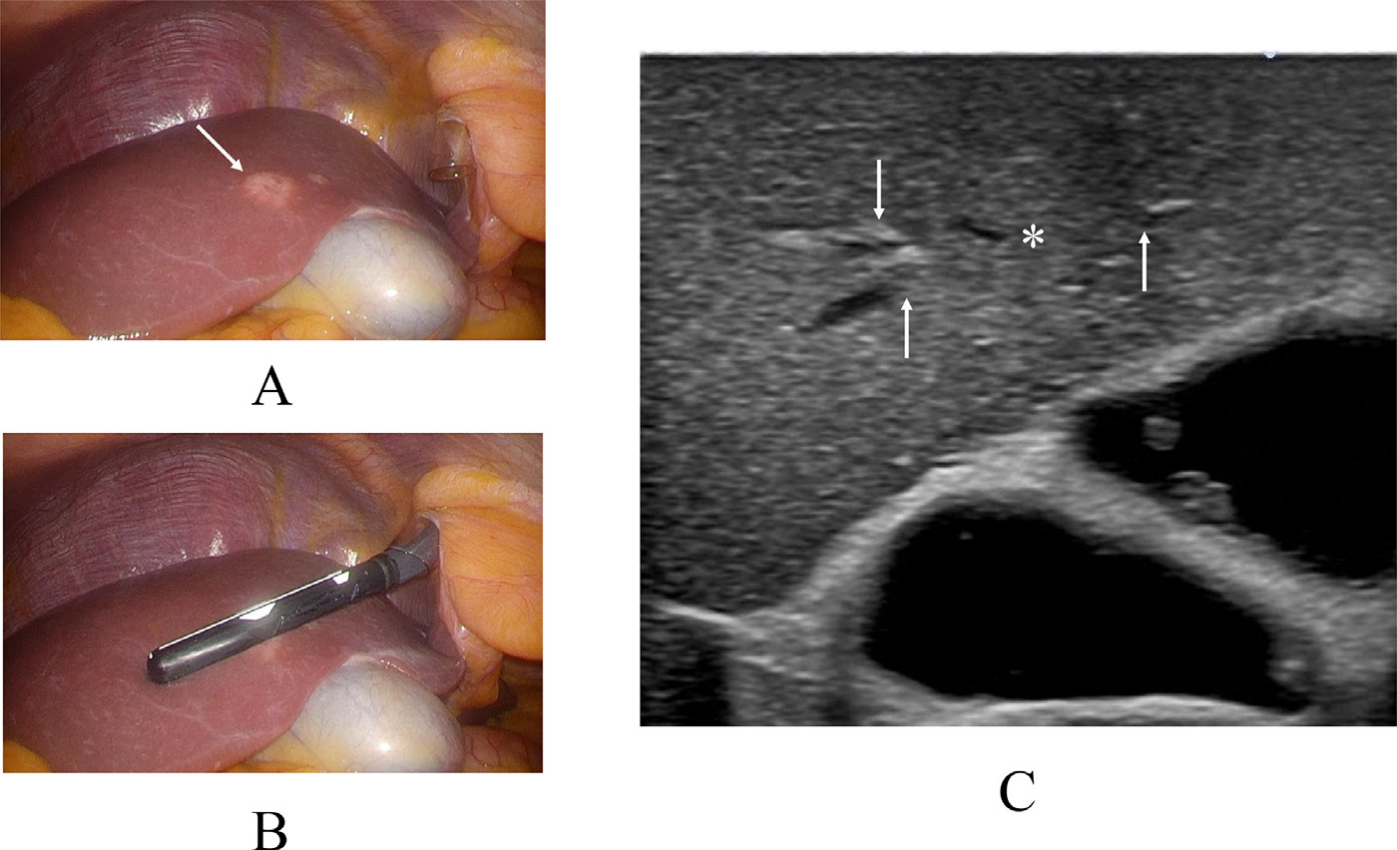
Intraoperative endoscopic and ultrasound findings. (A) The target tumor (arrow) was visible through the liver surface. (B) An endoscopic ultrasound probe was placed just on the tumor. (C) Intrahepatic mass (asterisk) was invisible on ultrasound. However, interruption of the peri-tumoral hepatic structures (arrows) highly suggested the presence of a hepatic mass.

**Fig. 4:**
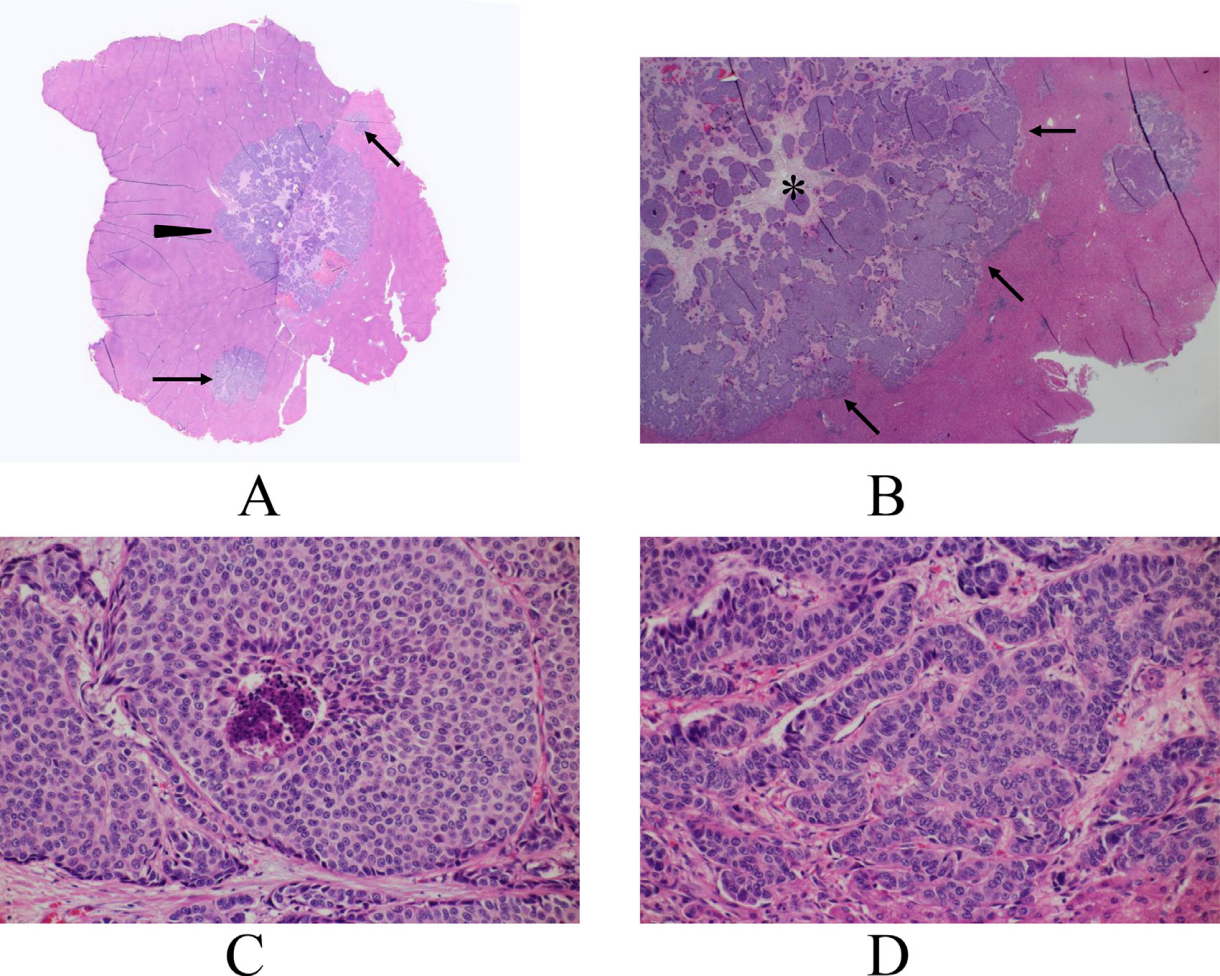
Pathological findings. (A) Low magnified view showed the main tumor (arrowhead) and the daughter nodules (arrows). (B) Magnified view showed that neither inflammatory cells nor fibrous tissues were present between the tumors and the surrounding normal hepatic cells (arrows). Small interstitial components (asterisk) were present between the tumor cell clusters. (C) Uniform tumor cells with little nuclear atypia grew in a solid fashion. (D) Tumor cells distributed in a cord-like fashion.

A 65-year-old woman on endocrine therapy after breast cancer surgery was referred to our hospital for detailed examination and treatment of a possible liver metastasis. PET / CT of the liver mass showed a maximal standardized uptake value (SUV max) of 14.3 in the liver segment 5 (Fig. 1). CT showed that the hepatic mass (figures not shown) had only faint ring enhancement. Magnetic resonance imaging (MRI) of the liver mass showed low signals on T1-weighted images, faint high signals on fat-suppressed T2-weighted images, nominal ring enhancement on early phases, retained very weak enhancement on late phases, and distinct low signals on hepatobiliary phases (Fig. 2). Ultrasound, however, failed to show any liver masses before operation. Impossible mass depiction on ultrasound made the patient undergo endoscopic laparoscopy for definitive diagnosis, which clarified an exposed liver tumor. However, even ultrasound by placing the ultrasound probe just on the exposed liver mass could not depict any mass images (Fig. 3). Macroscopic liver mass identification made us perform laparoscopic resection of the mass. Postoperative pathological study showed a well-circumscribed mass, 15 mm in size, with 3 daughter nodules, i.e., 1 mm, 2 mm, and 4 mm in size, around the main tumor. The tumors consisted of atypical cells growing in solid and trabecular fashions with little nuclear atypia, focal shelf-like arrangement of nuclei, a fine vascular network, small interstitial components between tumor cell clusters, and no peri-tumoral inflammatory cells/fibrous components (Fig. 4). Immunostaining showed that the tumors had arginase1, hepatocyte, TTF-1, CDX2, and PAX8 negativities, CD56, synaptophysin, and chromogranin positivities, and a Ki-67 labelling index of up to 15%. These pathological findings led to a diagnosis of metastatic grade 2 NET of an unknown primary focus. The patient recovered uneventfully and was discharged on the 5th day after the operation. The patient received somatostatin receptor scintigraphy to explore the primary NET postoperatively, unfortunately leading to no detection of the primary NET. The patient has been well for 8 months and is further scheduled to receive close follow-up surveillance with contrast-enhanced CT and ultrasound.

## Discussion

X-rays form images when irradiated X-rays are attenuated according to the X-ray attenuation coefficient of the target tissues or materials. However, in order to generate ultrasound tumor images, ultrasound waves must return to the ultrasound probe through some kind of mechanisms after hitting the target. There are 2 ways that ultrasound waves return to the ultrasound probe: reflection and back scattering [[4], [5], [6]].

The former makes the tumor shape and requires the presence of an interface, having a different acoustic impedance from those of the surrounding cells/tissues, that is much larger than the beam width of the ultrasound waves [4]. In this case, tumor cells directly contacted the normal liver cells without any interminglement of inflammatory cells or fibrous components pathologically and did not have large interfaces, capable of generating ultrasound wave reflection, at the tumor borders due to the presumed similar acoustic impedance between the tumor cells and the surrounding normal hepatic cells. This is the first reason why the tumor could not be visualized by ultrasound.

The latter determines the internal echoes of masses when ultrasound waves hit some kind of scattering bodies, being much smaller than the beam width of the ultrasound waves [5,6]. In this case, the cord-like arrangement of the tumor cells with little nuclear atypia closely resembled the palisading arrangement of normal hepatocytes. Furthermore, judged by the similarities in pathological morphology between the tumor cells and the surrounding normal hepatic cells, it seems unlikely that malignant cells growing in solid and trabecular patterns would show different internal echoes from those of hepatic cells arranged in a cord-like fashion. In addition, small interstitial components present between tumor cell clusters resembled central veins in the liver lobules. Therefore, NET cells presumably generated no distinguishable different internal echoes from those of the surrounding hepatic cells. This is the second reason why the tumor could not even be identified by ultrasound.

Many authors have reported the diagnostic difficulty both of primary hepatic NETs and metastatic NETs to the liver [[7], [8], [9], [10]]. Fatima et al. [7] reported a primary hepatic NET, 93 mm in size, and its difficulty of image diagnosis. They did not even provide any clear ultrasound images of the large primary NET but only described “mildly altered echotexture of liver” on ultrasound. Watanabe et al. [8] also reported a hepatic NET with multiple foci, 12cm in maximal size, but did not describe any ultrasound findings. These facts suggest that liver NETs, ​​whether primary or metastatic, are difficult to be clearly depicted by ultrasound.

The tumor had a fine vascular network, but no large vessels that could be identified by ultrasound. These findings can explain the faint early and retained weak enhancement observed both on CT and MRI. However, the hepatobiliary phase images of MRI using gadoxetic acid (EOB) showed very low signals, strongly suggesting the usefulness of EOB-MRI in diagnosing metastatic NETs to the liver.

Despite the small size of the main tumor, the SUV max of the tumor was very high as 14.1. This is likely due to the relatively high Ki-67 labeling index of the tumor cells and the lack of abundant stromal components such as mucus, fat, and fibrous components in the tumor. Since small hepatocellular carcinomas (HCCs) with early enhancement both on CT and MRI often have low SUV Max values due to the abundant phosphatase activities in well differentiated HCC cells. PET/CT, therefore, appears to be very useful for differentiating metastatic small NETs from early HCCs.

## Conclusion

Clinicians should be aware that ultrasound may fail to show mass images even of well-circumscribed metastatic NETs to the liver.

## Patient consent

Written informed consent was obtained from the patient for the publication of this case report and any accompanying images.
